# Two Italian Patients with *ELOVL4*-Related Neuro-Ichthyosis:  Expanding the Genotypic and Phenotypic Spectrum and Ultrastructural Characterization

**DOI:** 10.3390/genes12030343

**Published:** 2021-02-26

**Authors:** Andrea Diociaiuti, Diego Martinelli, Francesco Nicita, Claudia Cesario, Elisa Pisaneschi, Marina Macchiaiolo, Sabrina Rossi, Angelo Giuseppe Condorelli, Giovanna Zambruno, May El Hachem

**Affiliations:** 1 Dermatology Unit, Bambino Gesù Children’s Hospital, IRCCS, Piazza Sant’Onofrio 4, 00165 Rome, Italy; may.elhachem@opbg.net; 2 Genodermatosis Unit, Genetics and Rare Diseases Research Division, Bambino Gesù Children’s Hospital, IRCCS, Piazza Sant’Onofrio 4, 00165 Rome, Italy; agiuseppe.condorelli@opbg.net (A.G.C.); giovanna.zambruno@opbg.net (G.Z.); 3 Division of Metabolism, Bambino Gesù Children’s Hospital, IRCCS, Piazza Sant’Onofrio 4, 00165 Rome, Italy; diego.martinelli@opbg.net; 4 Unit of Neuromuscular and Neurodegenerative Disorders, Department of Neurosciences, Bambino Gesù Children’s Hospital, IRCCS, Piazza Sant’Onofrio, 4, 00165 Rome, Italy; francesco.nicita@opbg.net; 5 Laboratory of Medical Genetics, Department of Laboratories, Bambino Gesù Children’s Hospital, IRCCS, Piazza Sant’Onofrio, 4, 00165 Rome, Italy; claudia.cesario@opbg.net (C.C.); elisa.pisaneschi@opbg.net (E.P.); 6 Rare Diseases and Medical Genetics Unit, Bambino Gesù Children’s Hospital, IRCCS, Piazza Sant’Onofrio, 4, 00165 Rome, Italy; marina.macchiaiolo@opbg.net; 7 Pathology Unit, Department of Laboratories, Bambino Gesù Children’s Hospital, IRCCS, Piazza Sant’Onofrio, 4, 00165 Rome, Italy; sabrina2.rossi@opbg.net

**Keywords:** ELOVL4, ichthyosis, collodion baby, dysmorphism, epilepsy, developmental delay, visual evoked potentials, electron microscopy

## Abstract

Elongation of Very Long Chain Fatty Acid-4 (ELOVL4) is a fatty acid elongase responsible for very long-chain fatty acid biosynthesis in the brain, retina, and skin. Heterozygous mutations in *ELOVL4* gene cause Stargardt-like macular dystrophy and spinocerebellar ataxia type-34, while different homozygous mutations have been associated with ichthyosis, spastic quadriplegia, and mental retardation syndrome in three kindred. We report the first two Italian children affected with neuro-ichthyosis due to the previously undescribed *ELOVL4* homozygous frameshift variant c.435dupT (p.Ile146TyrfsTer29), and compound heterozygous variants c.208C>T (p.Arg70Ter) and c.487T>C (p.Cys163Arg), respectively. Both patients were born with collodion membrane followed by development of diffuse mild hyperkeratosis and scaling, localized erythema, and palmoplantar keratoderma. One infant displayed mild facial dysmorphism. They suffered from failure to thrive, and severe gastro-esophageal reflux with pulmonary aspiration. The patients presented axial hypotonia, hypertonia of limbs, and absent head control with poor eye contact from infancy. Visual evoked potentials showed markedly increased latency and poor morphological definition, indicative of alteration of the retro-retinal visual pathways in both patients. Ultrastructural skin examination revealed abnormalities of lamellar bodies with altered release in the epidermal granular and horny layer intracellular spaces. Our findings contribute to expanding the phenotypic and genotypic features of ELOVL4-related neuro-ichthyosis.

## 1. Introduction

Elongation of Very Long Chain Fatty Acid-4 (ELOVL4) is a fatty acid elongase involved in the biosynthesis of polyunsaturated and saturated very long-chain fatty acids (VLCFAs) [1]. The latter are precursors of both ω-O-acylceramides, which represent essential specific components of the stratum corneum lipid envelope in the epidermis, and sphingolipids in the brain. In turn, polyunsaturated VLCFAs are incorporated in phosphatidylcholine in retinal photoreceptors.

The first disease associated with heterozygous dominant-negative mutations in *ELOVL4* gene was a juvenile form of macular degeneration, known as Stargardt disease type 3 (MIM 600110) [2]. Subsequently, different *ELOVL4* heterozygous mutations were associated with autosomal dominant spinocerebellar ataxia type-34 (MIM 133190), a late onset cerebellar degenerative disease which may or not present erythrokeratodermia variabilis [3,4]. In parallel, homozygous mutations in *ELOVL4* gene were reported to cause a neuro-ichthyotic syndrome, named ichthyosis, spastic quadriplegia, and mental retardation (MIM 614457) in three kindred of different ethnicity [5,6]. Disease manifestations included congenital ichthyosis, epilepsy, spastic quadriplegia, and intellectual disability. 

We report the first two unrelated Italian patients affected with ichthyosis, spastic quadriplegia, and mental retardation due to novel biallelic mutations in *ELOVL4*. Our findings expand the phenotypic and genotypic characterization of this rare and severe syndrome. Moreover, skin ultrastructural features are described for the first time.

## 2. Materials and Methods

### 2.1. Histopathological and Ultrastructural Analyses

Skin biopsies were obtained after parents’ informed consent and processed for histopathological and ultrastructural examination, according to standard methods. 

### 2.2. Molecular Genetic Diagnosis 

Following informed consent, patient genomic DNA was extracted from peripheral blood using QIAsymphony DSP DNA Mini Kit (Qiagen, Hilden, Germany). Mutations were identified through Next Generation Sequencing (NGS) approach (NimbleGenSeqCap Target Enrichment—Roche, Madison, WI, USA; Twist Human Core Exome Kit - Twist Bioscience, San Francisco, CA, USA) and NextSeq550 or NovaSeq 6000 sequencing platforms (Illumina, San Diego, CA, USA). Identified variants were evaluated by VarSome [7] and classified according to the American College of Medical Genetics and Genomics (ACMG) guidelines [8].

## 3. Results

*Case 1*. A 2-year-old male baby, first child of healthy consanguineous parents (the father is first cousin of the maternal grandfather), was born at term by vaginal delivery. At birth, the patient was admitted to the neonatal intensive care unit due to respiratory failure and the presence of a thin membrane covering the skin. The collodion membrane peeled-off in the first days of life leaving a mild desquamation and erythematous patches. On day 2, the patient developed focal motor seizures, which were partially controlled by phenobarbital.

**Figure 1:**
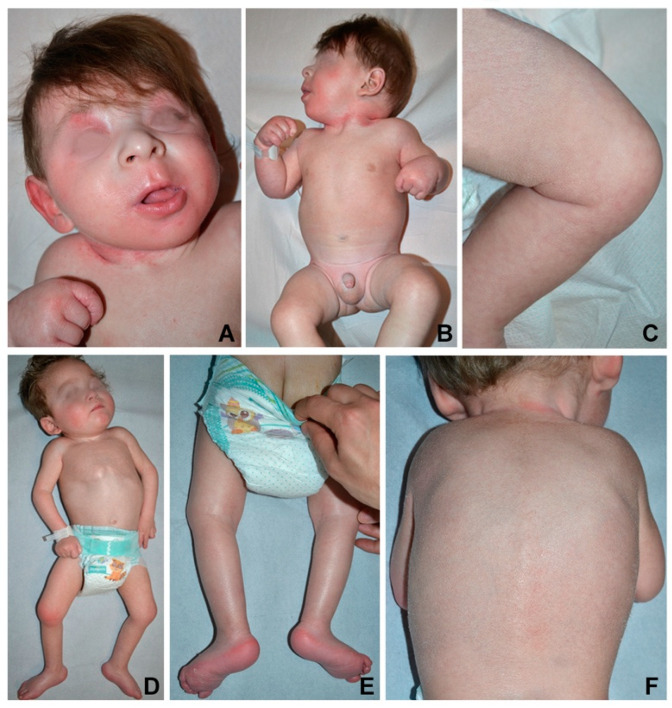
Clinical features: mild facial dysmorphism and erythema (**A**), proximal and distal limb hypertonia and neck erythema (**B**), slight thigh hyperkeratosis with fine whitish desquamation (**C**) in patient 1 at 7 months of age. Similar skin findings (**D**–**F**) and plantar keratoderma with erythema (**F**) are visible in patient 2 at 19 months of age. In the latter case, note also pectum carenatum and the dystonic posturing of the trunk, proximal limb hypotonia with frog-leg positioning and distal limb hypertonia with clenched fists (**D**).

The infant was referred to our hospital for psychomotor delay at the age of 3.5 months. Head circumference, length and weight were below the third percentile. Physical examination showed mild facial dysmorphism with flat nasal bridge, long philtrum and retrognathia (Figure 1A). Neurological examination revealed axial hypotonia with poor eye contact, absent head control, and hypertonia of upper and lower limbs (Figure 1B) with increased tendon reflexes. Ophthalmological evaluation did not show macular abnormalities. The skin was xerotic with fine whitish scales more evident on the scalp, upper back, diaper area and thighs, and erythema of palmoplantar surfaces, folds, neck, and face (Figure 1A–C).

**Figure 2:**
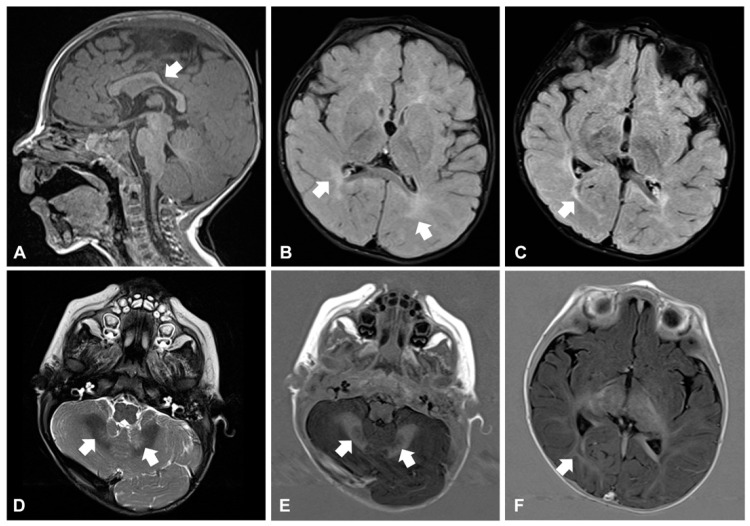
Brain magnetic resonance imaging in patient 1 at age 10 months. Corpus callosum is thin and mildly dysplastic at the level of isthmus (arrow in (**A**), sagittal view, T1-weighted image). The deep and periventricular white matter appears hyperintense compared to the grey matter (arrows in (**B**), axial view, FLAIR image), a similar abnormal high signal also involves the optic radiations (arrow in (**C**), axial view, FLAIR image). Finally, mild hyperintensity of the cerebellar dentate nuclei is visible (arrows in (**D**), axial view, T2 weighted image). T1-IR images confirm the involvement of dentate nuclei (arrows in (**E**), axial view) and optic radiations (arrow in (**F**), axial view).

Around the age of 10 months, he developed daily clusters of sudden and repeated flexions of arms and trunk, which were consistent with epileptic spasms. Electroencephalogram (EEG) showed hypsarrhythmia: West syndrome was diagnosed and therapy with vigabatrin started. Brain magnetic resonance imaging (MRI) revealed reduction and hyperintensity of the deep and periventricular white matter in the T2-weighted and FLAIR images, modestly hypodysplastic corpus callosum, and mild abnormal signal of the cerebellar dentate nuclei (Figure 2A–F). Electroretinogram was normal, while visual evoked potentials (VEP) revealed markedly increased latency and poor morphological definition, indicative of alteration of the retro-retinal visual pathways. The child also manifested feeding difficulties and recurrent respiratory infections with delayed growth. A gastroesophageal scintigraphy showed markedly delayed gastric emptying and severe gastroesophageal reflux (GER) associated with pulmonary aspiration, confirmed by salivogram. Percutaneous endoscopic jejunostomy was performed to ensure sufficient nutrient intake.

*Case 2.* A 3-year-old male baby, third child of healthy non-consanguineous parents, was born at term by repeat cesarean section. A collodion membrane was present at birth requiring hospitalization in neonatal intensive care unit. The membrane rapidly shed leaving ear deformities and erythema, which also resolved. From the second month of life the child presented focal motor seizures treated with topiramate and clobazam. In addition, jerky movements and facial hypomimia were noticed. Brain MRI showed mildly delayed myelination, enlarged occipital horns of the lateral ventricles, hypoplasia of corpus callosum and widened subarachnoid spaces. EEG revealed poorly organized background activity with recurrent slow anomalies and spike-and-waves complexes on the bilateral posterior regions. The infant also had recurrent respiratory infections and severe GER, which required nasogastric tube feeding.

The infant was referred to us at 4 months of age. Head circumference was at the third percentile, while length and weight were below it. Physical examination showed mild diffuse hyperkeratosis with fine whitish desquamation, palmoplantar keratoderma as well as inguinal, axillary, and neck erythema (Figure 1D–F). Neurological examination revealed poor eye contact, reduced spontaneous movements, axial hypotonia with no head control, dystonic posturing of the trunk and distal limb hypertonia (Figure 1D).

Gastroesophageal scintigraphy showed delayed gastric emptying and severe GER associated with pulmonary aspiration, confirmed by salivogram. Thus, percutaneous endoscopic gastrostomy was placed to improve nutritional status and avoid aspiration. During follow-up, an ophthalmological evaluation at age 2.5 showed bilateral optic atrophy and complete absence of light and threat reflexes as well as lack of visual fixation and ability to follow objects. VEP revealed markedly increased latency and poor morphological definition. Focal seizures recurred with daily frequency requiring treatment with carbamazepine and clonazepam. Trihexyphenidyl treatment resulted in reduction of dystonic posturing.

**Figure 3:**
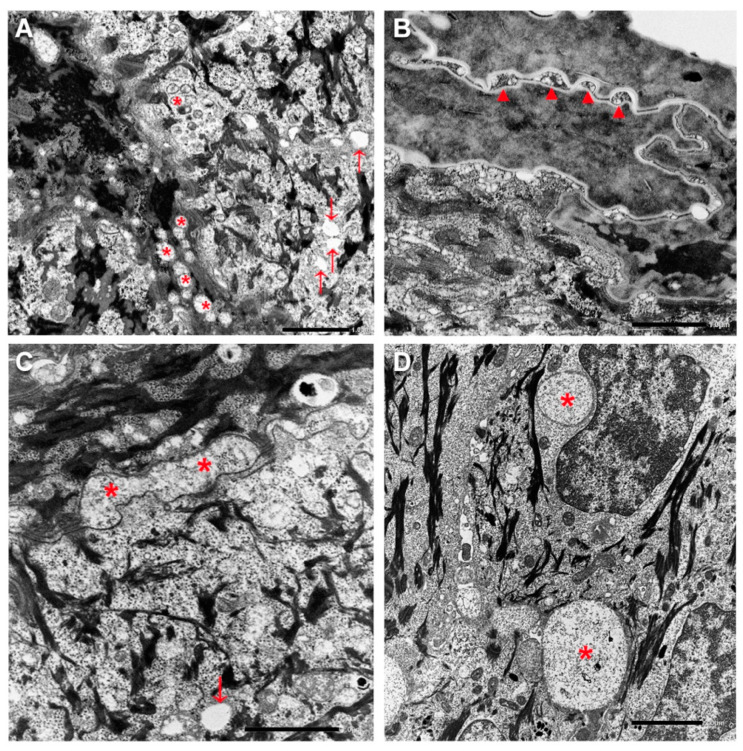
Ultrastructural features. Abnormal lamellar bodies with disorganized and disrupted lamellar content (asterisks) and some empty vacuoles (arrows) within the upper spinous and granular layer in patient 1 (**A**). Dilated horny layer intercellular spaces filled with disorganized lamellar structures (arrow) in patient 1 (**B**). Dilated intercellular spaces within the granular layer containing amorphous material in patient 2 (asterisks) and empty vacuoles (arrow) (**C**). Large membrane-bound cytoplasmic vacuoles (asterisks) with a finely granular content within the lower epidermis in patient 2 (**D**). Ba r= 1 µm in (**A**–**C**), 2 µm in (**D**).

*Histopathological and ultrastructural findings.* Histopathological examination of lesioned skin punch biopsies from left thigh showed modest epidermal acanthosis and papillomatosis, a well-represented granular layer (GL) and compact hyperkeratosis with isolated parakeratotic cells in both patients. In addition, several irregularly scattered cytoplasmic vacuoles were visible within the basal and suprabasal epidermal layers (Supplementary Figure S1). Ultrastructural examination revealed abnormal lamellar bodies of variable size with disrupted and disorganized lamellar content in the epidermal spinous and granular layers (Figure 3A). Additionally, small empty vacuoles were seen in the GL. Intercellular spaces of the upper granular and horny layers were frequently dilated and filled with disorganized lamellar structures and amorphous material (Figure 3B,C). A few corneocytes containing nuclear remnants and inhomogeneous keratin matrix were observed. Finally, several membrane-bound vacuoles, ranging from 0.5 to 5 μm in diameter and containing a finely granular material, were detected in the lower epidermal layers (Figure 3D).

**Figure 4:**
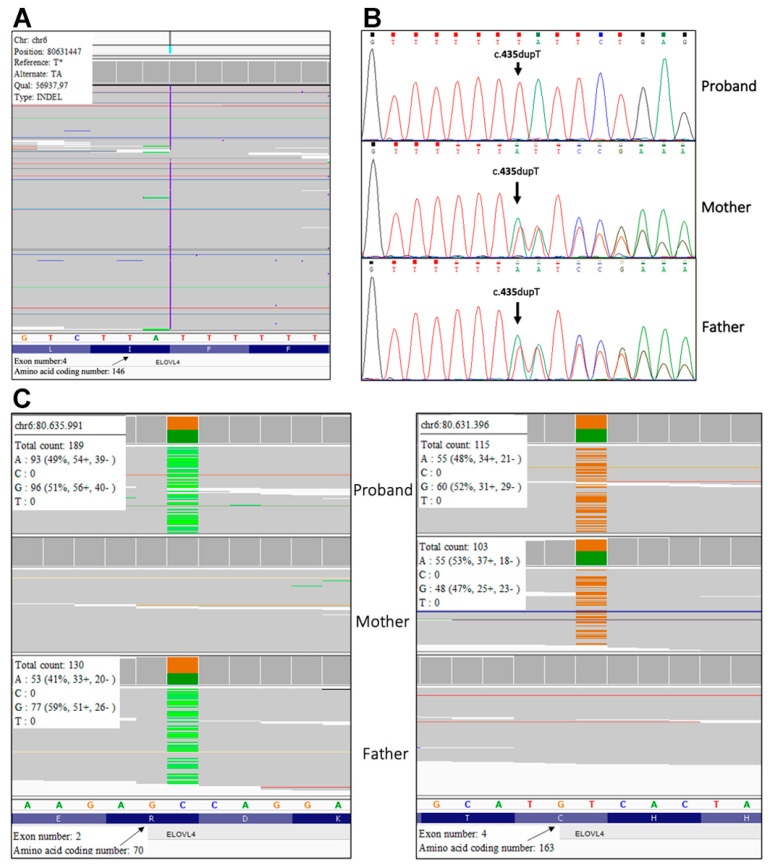
Molecular genetic testing. Next generation sequencing (NGS) singleton analysis shows the *ELOVL4* (NM_022726.3) variant c.435dup, p.(Ile146Tyrsfs*29) at the homozygous state in patient 1 (**A**), and Sanger analysis confirms the presence of the same mutation in the homozygous proband and his heterozygous parents (**B**); trio-based NGS analysis identifies the *ELOVL4* paternal variant c.208C>T, p.(Arg70*) (left image) and the maternal variant c.487T>C, p.(Cys163Arg) (right image) in patient 2 (**C**).

*Mutation analysis.* Molecular genetic testing performed with a customized ichthyosis gene panel revealed the homozygous frameshift sequence variant c.435dup, p.(Ile146Tyrsfs*29) in exon 4 of *ELOVL4* (NM_022726) gene in patient 1 (Figure 4A). Sanger sequencing confirmed the mutation in the proband and revealed that his healthy parents are heterozygous (Figure 4B). The variant has not been previously reported and is not annotated in GnomAD database of human variations (https://gnomad.broadinstitute.org/). It was considered pathogenic according to ACMG guidelines as (i) it is a null variant in a gene for which loss-of-function is a known disease mechanism (PVS1), (ii) it is novel (PM2), and (iii) computational evidences support a deleterious effect on the gene product (PP3).

In patient 2, trio-based whole exome analysis identified the paternal variant c.208C>T, p.(Arg70*) (rs750620675) and the maternal variant c.487T>C, p.(Cys163Arg) in exon 2 and 4 of *ELOVL4* gene, respectively (Figure 4C). The paternal variant p.(Arg70*) is found in the GnomAD database with an allele frequency of 0.000003980, while the maternal missense variant p.(Cys163Arg) has not been previously reported. According to ACMG guidelines variant p.Arg70Ter was considered pathogenic as it is a null variant (PVS1), it is detected at extremely low frequency in controls (PM2), and computational evidences support a deleterious effect on the gene product (PP3). The variant p.(Cys163Arg) was classified as likely pathogenic because it is not found in GnomAD database (PM2), computational evidence supports his deleterious effect (PP3) and it is detected in *trans* with a pathogenic variant for a recessive disorder (PM3). 

## 4. Discussion

Neurocutaneous disorders presenting with ichthyosis, also referred to as neuro-ichthyotic syndromes, are a large group of clinically and genetically heterogeneous conditions mostly manifesting at birth with skin signs which range from collodion baby to scales and/or erythema [9,10,11]. Neurological signs and symptoms can appear from the first year through early adulthood. They comprise variable combinations of global developmental delay, spasticity or hypotonia, epilepsy, sensorineural deafness, visual impairment and neuropathy. Involvement of additional organ/systems, from kidney to liver, gastrointestinal and endocrine systems, is frequent. Neuro-ichthyotic syndromes are caused by a wide range of genetic defects primarily affecting lipid metabolism, but also glycoprotein synthesis, peroxisomal function, or intracellular vesicle trafficking. 

Sjögren–Larsson’s syndrome is the prototypic neuro-ichthyotic syndrome presenting with ichthyotic skin features, spasticity, and mental retardation. Disorders with similar phenotype include cerebral dysgenesis-neuropathy-ichthyosis-palmoplantar keratoderma (CEDNIK) syndrome, due to mutations in *SNAP29* involved in intracellular vesicle trafficking, and two syndromes due to mutations in the fatty acid elongases, *ELOVL1* and *ELOVL4*, acting in the biosynthesis of VLCFAs. Heterozygous mutations in the *ELOVL1* gene cause a recently described autosomal dominant syndrome, named IKSHD for ichthyotic keratoderma, spasticity, hypomyelination, and dysmorphic facial features [12]. On the other hand, *ELOVL4* biallelic mutations are responsible for ichthyosis, spastic quadriplegia, and mental retardation syndrome. 

We describe the first two Italian patients with neuro-ichthyotic syndrome due to *ELOVL4* biallelic mutations. To date, only two sporadic cases from Saudi Arabia and India, respectively, and a Pakistani family have been reported [5,6]. Both our patients were born as a mild collodion baby similar to a single previous case [5]. Ichthyosis was characterized by slight hyperkeratosis, fine whitish scaling, and flexural erythema, consistent with previous reports [5,6]. In addition, one patient developed palmoplantar keratoderma, a feature not yet described. The mild skin phenotype was associated with major neurological manifestations comprising early-onset drug-resistant epilepsy, intellectual disability and spastic-dystonic tetraparesis, in line with the severe neurological impairment observed in the first two cases described [5]. However, only one of the three affected Pakistani siblings had typical neurological manifestations, indicating that intra-familiar phenotypic variability can occur [6]. Neuro-imaging is available only for one previous patient, who presented a severely delayed myelination and brain atrophy at 6 months of age [5]. Our findings seem to support the presence of white matter involvement. However, both our patients had a single brain MRI performed in the first year of age, thus limiting accurate evaluation of the myelination process. Nevertheless, the involvement of optic radiations and cerebellar dentate nuclei seen in patient 1 at 10 months is indicative of altered myelination since these structures are normally myelinated around 1 year of age. Ophthalmological examination of our patients confirmed lack of macular changes specific for Stargardt disease type 3 [5,6]. On the other hand, in both our patients VEP indicated alteration of the retro-retinal visual pathways. Interestingly, IKSHD syndrome due to *ELOVL1* dominant mutations presents progressive loss of visual acuity and peripheral vision secondary to optic atrophy [12]. Furthermore, marked growth delay was present in our patients as well as in the first two described cases [5]. Our patient 1 also showed mild facial dysmorphism, a feature not previously reported. Finally, our cases presented severely delayed gastric emptying, as frequently observed in severe encephalopathies.

Ultrastructural examination showed remarkably similar findings in both children, consisting of abnormalities of lamellar bodies with altered release in the intracellular spaces. These features are in line with those observed in the *Elovl4* knock-out mice and with the defective synthesis of VLCFAs and consequently of ω-O-acylceramide due to ELOVL4 functional impairment [13]. Comparable ultrastructural findings have also been reported in other congenital ichthyoses due to mutations in enzymes, in particular PNPLA1 and ABHD5, involved in the biosynthesis of ω-O-acylceramide [14,15,16]. In addition, in our patients, several membrane-bound vacuoles were detected by light and electron microscopy within the keratinocytes of lower epidermis. Further studies are needed to establish their nature and content, and whether they represent a recurrent feature in ELOVL4 neuro-ichthyosis.

All *ELOVL4* mutations identified to date in ichthyosis, spastic quadriplegia, and mental retardation were homozygous null mutations as in our patient one. The Pakistani family with variable expressivity carried the most upstream premature termination codon (PTC) in exon one (p.Y26Ter), while the two original patients with severe phenotype carried a nonsense and a frameshift mutation in exon five and six, respectively [5,6]. Thus, loss of function has been suggested as the mechanism underlying disease pathogenesis. Interestingly, we describe for the first time a missense mutation, p.(Cys163Arg), found in compound heterozygosity with a PTC in patient 2. The missense mutation p.(Cys163Arg) is located next to the elongase catalytic site (aa 158–162) in a highly conserved region among vertebrates, suggesting that it impairs enzymatic function and/or destabilizes protein structure resulting in degradation [1]. Finally, since the 42-year-old mother carrying the missense p.(Cys163Arg) is healthy, it is highly unlikely that the amino acid substitution behaves as a dominant mutation similar to other missense located in exon 4 which are responsible for spinocerebellar ataxia-34.

## 5. Conclusions

In conclusion, we highlighted novel genotypic and phenotypic features in ELOVL4-related neuro-ichthyotic syndrome contributing to expand the characterization of this very rare and severe disorder, and described ultrastructural epidermal abnormalities recurrent in ichthyoses due to defects of ω-O-acylceramide biosynthesis.

## Data Availability

The data presented in this study are available on request from the corresponding author. The data are not publicly available due to privacy reasons.

## Supplementary Materials

The following are available online at https://www.mdpi.com/2073-4425/12/3/343/s1, Figure S1: histopathological findings.
