# Supplementary material for: Two Italian Patients with ELOVL4-Related Neuro-Ichthyosis: Expanding the Genotypic and Phenotypic Spectrum and Ultrastructural Characterization
Source: Genes (Basel). 2021 Feb 26;12(3):343. doi: 10.3390/genes12030343 (PMC7996761; doi:10.3390/genes12030343)
Supplement: Supplementary file 1 [file genes-12-00343-s001.zip › Supplementary Figure S1.docx]

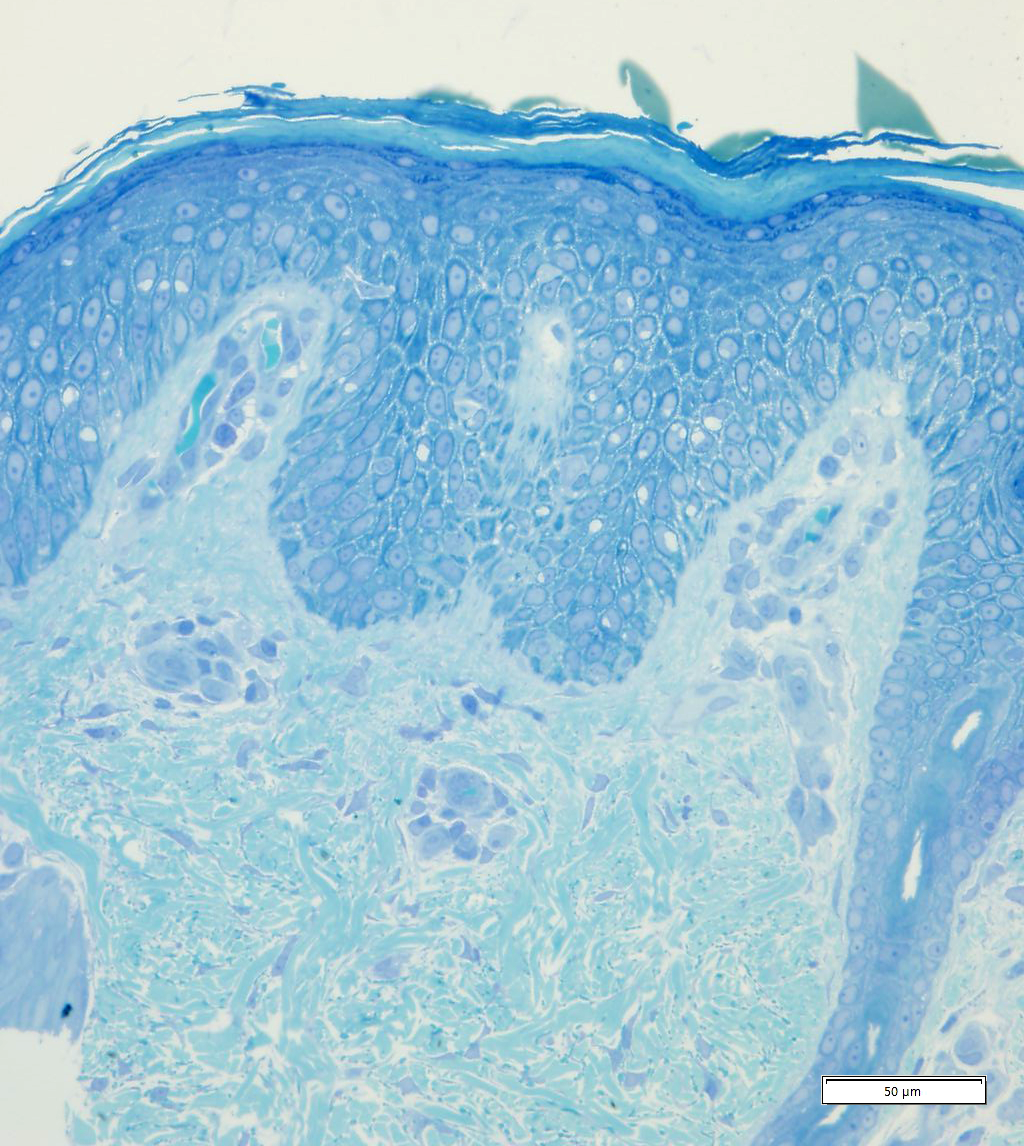


**Supplementary Figure S1.** Semithin section showing epidermal acanthosis and papillomatosis, mild hypergranulosis and compact hyperkeratosis. Several cytoplasmic empty vacuoles of variable size are visible within the basal and suprabasal epidermal layers (Azur II-methylene blue, bar 50 µm)**.**
