# Supplementary figures and images for: Two Italian Patients with ELOVL4-Related Neuro-Ichthyosis: Expanding the Genotypic and Phenotypic Spectrum and Ultrastructural Characterization
Source: Genes (Basel). 2021 Feb 26;12(3):343. doi: 10.3390/genes12030343 (PMC7996761; doi:10.3390/genes12030343)

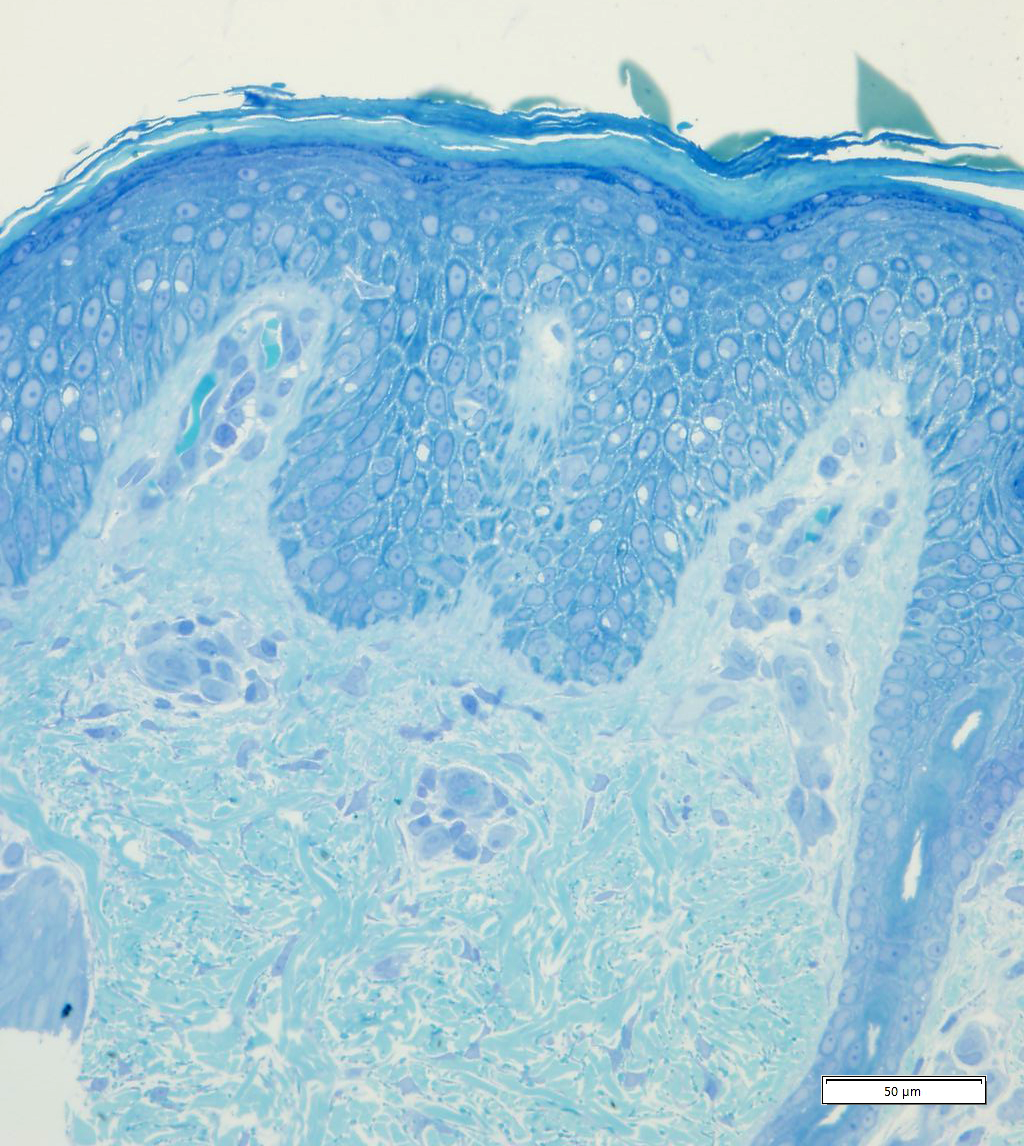

Supplement: Supplementary file 1 [file genes-12-00343-s001.zip › Figure S1.tif]
